# First-in-human clinical trial of personalized neoantigen vaccines as early intervention in untreated patients with lymphoplasmacytic lymphoma

**DOI:** 10.21203/rs.3.rs-3315017/v1

**Published:** 2023-09-21

**Authors:** Larry Kwak, Szymon Szymura, Lin Wang, Tiantian Zhang, Soung-chul Cha, Zhenyuan Dong, Aaron Anderson, Elizabeth Oh, Vincent Lee, Zhe Wang, Sapna Parshottham, Sheetal Rao, Jasper Olsem, Brandon Crumpton, Hans Lee, Elisabet Manasanch, Sattva Neelapu, Sheeba Thomas

**Affiliations:** City Of Hope National Medical Center; City of Hope, Beckman Research Institute, Toni Stephenson Lymphoma Center; City of Hope, Beckman Research Institute, Department of Computational and Quantitative Medicine; City of Hope, Beckman Research Institute, Toni Stephenson Lymphoma Center; City of Hope, Beckman Research Institute, Toni Stephenson Lymphoma Center; City of Hope National Medical Center; City of Hope National Medical Center; City of Hope National Medical Center; City of Hope National Medical Center; City of Hope National Medical Center; MD Anderson Cancer Center; MD Anderson Cancer Center; MD Anderson Cancer Center; MD Anderson Cancer Center; The University of Texas MD Anderson Cancer Center; The University of Texas MD Anderson Cancer Center; The University of Texas MD Anderson Cancer Center; The University of Texas MD Anderson Cancer Center

## Abstract

Lymphoplasmacytic lymphoma (LPL) is an incurable low-grade B-cell lymphoma of the bone marrow. Despite a cumulative risk of progression, there is no approved therapy for patients in the asymptomatic phase. We conducted a first-in-human clinical trial of a novel therapeutic DNA idiotype neoantigen vaccine in nine patients with asymptomatic LPL. Treatment was well tolerated with no dose limiting toxicities. One patient achieved a minor response, and all remaining patients experienced stable disease, with median time to disease progression of 61+ months. Direct interrogation of the tumor microenvironment by single-cell transcriptome analysis revealed an unexpected dichotomous antitumor response, with significantly reduced numbers of clonal tumor mature B-cells, tracked by their unique BCR, and downregulation of genes involved in signaling pathways critical for B-cell survival post-vaccine, but no change in clonal plasma cell subpopulations. Downregulation of HLA class II molecule expression suggested intrinsic resistance by tumor plasma cell subpopulations and cell-cell interaction analyses predicted paradoxical upregulation of IGF signaling post vaccine by plasma cell, but not mature B-cell subpopulations, suggesting a potential mechanism of acquired resistance. Vaccine therapy induced dynamic changes in bone marrow T-cells, including upregulation of signaling pathways involved in T-cell activation, expansion of T-cell clonotypes, increased T-cell clonal diversity, and functional tumor antigen-specific cytokine production, with little change in co-inhibitory pathways or Treg. Vaccine therapy also globally altered cell-cell communication networks across various bone marrow cell types and was associated with reduction of protumoral signaling by myeloid cells, principally non-classical monocytes. These results suggest that this prototype neoantigen vaccine favorably perturbed the tumor immune microenvironment, resulting in reduction of clonal tumor mature B-cell, but not plasma cell subpopulations. Future strategies to improve clinical efficacy may require combinations of neoantigen vaccines with agents which specifically target LPL plasma cell subpopulations, or enable blockade of IGF-1 signaling or myeloid cell checkpoints.

## Introduction

LPL is an incurable low-grade B-cell lymphoma, characterized by the presence of clonal lymphoplasmacytic cells infiltrating the bone marrow as the primary organ, and a serum monoclonal protein. IgM-secreting LPL, known as Waldenström macroglobulinemia (WM), is the most common subtype ^1^. In the absence of end organ damage, patients are considered to have smoldering phase disease. There is no approved standard therapy for smoldering LPL, and patients are generally managed by active surveillance ^1^. Accordingly, the availability of a well-tolerated therapeutic agent that would enable early intervention to delay progression to symptomatic phase disease and other complications, without inducing cross-resistance to subsequent chemotherapies, is desirable.

Tumor neoantigens recognized by T cells are emerging as targets for the design of cancer vaccines, and initial clinical trials have demonstrated both safety and efficacy ^2–4^. One of the first neoantigens tested was the unique B-cell receptor generated by clonal rearrangement of Ig variable region gene sequences by lymphoma cells, rather than somatic mutation, referred to as idiotype ^5–8^ . Idiotype peptides were the dominant neoantigens eluted from HLA molecules in human lymphomas ^9,10^. Therapeutic idiotype vaccines have been shown to elicit robust CD8 + T-cell immunity in humans, and one randomized, controlled clinical trial demonstrated improved disease-free survival in a minimal residual disease setting following induction chemotherapy in follicular lymphoma ^11–15^.

Here, in a first-in-human clinical trial we tested a novel DNA vaccine platform, encoding the autologous LPL-derived Ig single chain variable fragment (scFv) fused to human chemokine CCL20 (macrophage inflammatory protein-3, MIP-3a), which was designed to trigger T-cell immunity by targeting delivery of the expressed fusion protein to antigen presenting cells ^16,17^ in smoldering LPL patients.

## Results

### Patient characteristics

A total of nine patients were enrolled and treated on trial, three in the 500μg cohort and six in the 2500μg cohort. The baseline characteristics of patients are described in Table 1. The median age of all patients was 67, and the majority were male (78%). Of eight patients with baseline gene mutation data available, six had MYD88 mutations; of these, one had a CXCR4 WHIM mutation. The median time from diagnosis of asymptomatic LPL to first vaccination was 2.2 years.

### Safety, tolerability, and response assessment

All patients successfully completed planned therapy. No patients in either cohort experienced dose limiting toxicities (DLTs) or Grade 4 adverse events (AEs). Ten months after the last vaccination, LPL-005 developed a grade 3 non-malignant pleural effusion, grade 1 pericardial effusion, and leukocytopenia, accompanied by an increase in rheumatoid factor (23.1 IU/mL [normal range 0.0–15.9]) and an ANA titer of 1:80; all findings resolved within 2 months. Grade 1–2 AEs occurring in 3 or more patients were leukopenia, nausea, myalgias, fatigue, diarrhea, anemia, hyperglycemia, and increased creatinine. Details are provided in Table 2.

**Figure 1:**
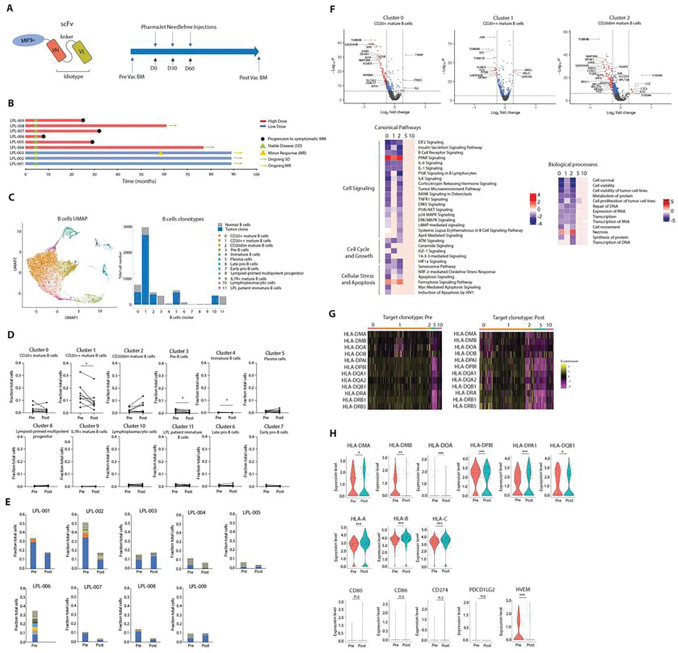
Single cell RNA-seq analysis reveals vaccine-associated reduction of LPL B-cell but not plasma cell-like subpopulations in the bone marrow tumor microenvironment. (**A**) Schematic of individualized chemokine-idiotype DNA vaccines and vaccine treatment schedule. (**B**) Swimmer plot illustrating clinical responses of each patient (patients are designated as LPL-001 through –009) (**C**) UMAP of B-lineage cell populations extracted the from total dataset. 12 clusters were generated (left). Total cell numbers per cluster are shown for normal B-lineage cells and specifically, tumor B cells, based on known idiotype sequences (right). (**D**) Cell frequencies by total B-lineage cluster for each patient in paired pre- and post-vaccine bone marrow samples. Paired student t-test was used (**E**) Cell frequencies of major BCR clonotypes in all B-cell clusters pre- and post-vaccine for each patient. Blue = dominant tumor idiotype clone isolated for vaccine production; all other colors indicate normal B-cell clonotypes. (**F**) Pooled data from all patients. Volcano plots of differentially expressed genes (adjusted p-value > 0.05) pre- vs. post-vaccine for the major B-lineage clusters containing tumor cells (clusters 0–2, top). Ingenuity Pathway Analysis (Qiagen IPA) based on differentially expressed genes (z-score > 2, adjusted log p-value >1.3) of canonical pathways (bottom left) and biological processes (bottom right) contrasting tumor mature B-cell (0–2) and plasma cell-like clusters (5 and 10), respectively. (**G**) Heatmap of selected HLA class II gene expression by idiotype clonal tumor cells pre- and post-vaccine relative to total cells and grouped by B-lineage cluster. (**G**) Violin plots of expression of HLA family genes and co-inhibitory ligands by idiotype clonal tumor cells pre- and post-vaccine in cluster 2 only. Two-sided Wilcoxon test was used. *P ≤ 0.05, **P ≤ 0.01, ***P ≤ 0.001, and ****P ≤ 0.0001

Using response criteria from the 6th International WM Workshop Consensus Panel, LPL-003 achieved a minor response (MR). The best response for the remaining eight patients was stable disease (SD) (Fig. 1B). After a median follow-up period of 77 months for all patients, four patients have experienced progression to symptomatic WM, requiring initiation of systemic therapy (LPL-005, -006, -007, and - 009) at 29, 8, 32, and 25 months, respectively. LPL-006 experienced early disease progression and was lost to follow up 8.8 months after last vaccination, before a post-vaccine bone marrow sample could be obtained. All remaining patients are known to be alive.

### Reduction in clonal tumor subpopulations and their gene expression pathways after vaccination in the mature B-cell, but not in the LPL plasma cell-like compartment

To interrogate vaccine-induced changes directly in the tumor microenvironment, bone marrow samples were obtained a median of 3 months (range 1–13 months) after vaccine treatment from all nine patients, except patient LPL-006. We performed single cell RNA-seq analysis on matched pre- and post-vaccine bone marrow samples, paired with matched single cell BCR and TCR sequencing (Fig. S1B-F). To analyze specific changes in LPL cells following vaccination we separated and re-clustered heterogeneous B-lineage populations and obtained a total of 12 clusters based on differential gene expression (Fig. 1C, Fig. S2A). To specifically identify clonal tumor cells across various clusters we matched single cell BCRs with the previously identified unique tumor idiotype (Ig VH and VL CDR3) sequences used for manufacturing individualized therapeutic vaccines for each patient. LPL is known to consist of distinct clonal B-cell- and plasma cell-like subpopulations^18,19^. Clusters 0, 1 and 2, representing mature B cells, were comprised almost entirely of the tumor clonotype, with cluster 1 being the most abundant (Fig. 1C, right). Plasmablast-like and mature plasma cells (clusters 5 and 10, respectively) were less abundant but also contained relatively high proportions of tumor clonotypes (Fig. 1C, right). Analysis of paired total B-lineage cells pre- vs. post-vaccine showed significantly reduced frequencies post-vaccine for B-cell cluster 1 but not for the plasma cell-like clusters (Fig. 1D). This reduction in B-cell frequencies in cluster 1 was entirely attributable to a specific reduction in the tumor clonotype. This reduction in tumor cell clonotype frequencies was observed in all except for three evaluable patients (Fig. 1E).

Concomitant changes in global gene expression patterns in tumor cells were associated with the reduction of the tumor mature B-cell compartment post-vaccine. Differential gene expression analysis of each of the relevant B-lineage cell clusters revealed a pattern of significant gene downregulation following vaccine in clusters 0, 1 and 2 (Fig. 1F, top). Among the top downregulated genes were FOS, JUN, ATF3, ATF4, NFKBIA and MAP3K8 which are essential for the growth of B lymphocytes ^20,21^ as well as proteins of the EIF (eukaryotic initiation factor) family (EIF4A1 ^22^, EIF4A2, EIF4A3), GADD34 ^23^, ribosomal protein L family (RPL4, RPL9, RPL13, RPL21, RP23, PRL27, RPL37, RPL38, RPL10A) and ribosomal proteins family (RPS2, RPS6, RPS9, RPS11, RPS16, RPS20, RPS26, RPS27) which are essential for lymphoma cell proliferation and protein synthesis ^24^ (Fig. 1F, top). Furthermore, pathway analysis based on differentially expressed genes identified signaling pathways significantly reduced post vaccine known to be critical for B-cell survival including IL-1^25^, IL-6^26^, IGF-1^27^ and APRIL ^28^ (Fig. 1F, bottom left). BCR ^29^, PI3K/AKT ^30,31^ and ERK/MAPK, which are involved in survival-promoting signaling by mutant MYD88 in WM cells, were also significantly downregulated ^32,33^. Conversely, PPAR signaling, which is known to promote tumor cell apoptosis ^34^, and ferroptosis cell death pathways ^35^ were both upregulated by these clusters. Finally, the analysis predicted overall downregulation of biological processes (z-score > 2, adjusted log p-value > 1.3) including cell survival, viability, proliferation, protein synthesis and RNA transcription and upregulation of necrosis (Fig. 1F, bottom right). Notably, no global changes were inferred for corresponding plasma cell-like clusters. These observations suggest that tumor subpopulations of LPL within a single patient may be dichotomous in their response to therapeutic vaccine treatment, with mature B-cell subpopulations more susceptible than plasma cell-like cells.

A well-described mechanism of tumor cell resistance to T-cell-mediated killing is the downregulation of expression of HLA family genes, particularly HLA class II genes ^36–40^. To investigate this possibility, we compared expression of HLA family genes in tumor cells in relevant B- and plasma-cell clusters. Consistent with previous reports we observed downregulation of HLA class II family (HLA-DMA, HLA-DMB, HLA-DPA1, HLA-DPB1, HLA-DQA2) gene expression in clusters 5 and 10 containing plasmablast-like and plasma cells in both pre- and post-vaccine samples, but not in B-cell clusters 0 and 1 (Fig. 1G) ^41,42^. Interestingly, there was also a trend towards downregulation of expression of HLA class II (but not HLA class I) genes post-vaccine, compared with pre-vaccine in B-cell cluster 2 tumor cells (Fig. 1GH). In contrast, no significant changes were observed in tumor expression of T-cell checkpoint ligands, including PDL1 (CD274), and PDL2 (PDCD1LG2) (Fig. 1H and not shown). We also observed no significant differences in expression of genes of the death receptor family among clonal tumor B-cell or plasma cell-like clusters post-vaccine (Fig S1G) ^43^. Taken together these observations suggest that plasma-cell subpopulations of clonotypic tumor cells in LPL may exhibit immune evasion to our vaccine therapy by downregulating expression of HLA genes, rather than by activation of T-cell immune checkpoints.

### Paired single cell transcriptomics reveals dynamic changes in T cells in the tumor microenvironment following vaccine treatment.

**Figure 2:**
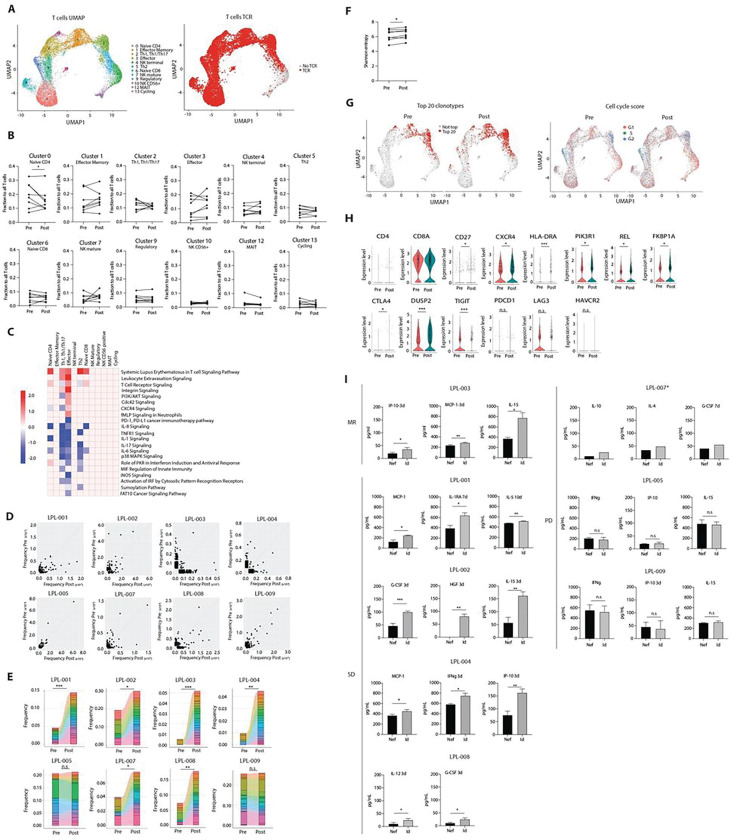
Paired single cell TCR-seq reveals T-cell clonal expansion and activation in the tumor microenvironment following vaccine treatment. (**A**) UMAP of T-cell subpopulations extracted the from the total dataset. A total of 12 clusters were generated with unique phenotypes (left). UMAP of T-cells highlighting cells with single cell TCR sequences detected (right). (**B**) T-cell frequencies pre- and post-vaccine for individual patients by T-cell cluster. Paired student t-test was used (**C**) Heatmap showing selected immune signaling pathways post vs pre vaccine (adjusted log p-value > 1.3) based on significantly differentially expressed genes for each indicated T cell subpopulation using IPA software (Qiagen). Pooled data from all patients. (**D**) Scatterplots of TCR clonotype frequencies pre-vaccine (y-axis) and post-vaccine (x-axis) for each patient. (**E**) Frequencies of the 20 most prevalent T-cell clonotypes identified within the post-vaccination T-cell repertoire, compared with their matching clonotype pre-vaccine. Paired student t-test was used. (**F**) Shannon entropy of TCR clonotype repertoires in paired samples pre. vs post vaccine for each patient. Paired student t-test was used (**G**) UMAPs of pooled T-cell subpopulations in pre- and post-vaccine samples with highlighted cells in the 20 most prevalent post-vaccine clonotypes (left panels) and the cell cycle phases inferred from gene expression signatures using Seurat package (right panels). (**H**) Violin plots of expression levels of selected gene markers by the top 20 T-cell clonotypes pre- vs. post-vaccine. Two-sided Wilcoxon test was used. (**I**) Functional tumor idiotype-specific T-cell responses post-vaccination. Bone marrow samples from each patient were enriched for T cells by negative selection and then 2.5×10^5^ cells per well were stimulated in triplicate with autologous immortalized B cells as APCs (2.5×10^5^ cells / well) which had been transfected with either patient-specific tumor idiotype (Ig VH and VL genes) or HIV Nef as a negative control. Supernatants were harvested 3, 7, or 10 days later and analyzed by multiplex cytokine assay. Representative cytokines are shown for each patient. MR – minor response, SD – stable disease, PD – progressive disease. Two-sided student t-test was used. *P ≤ 0.05, **P ≤ 0.01, and ***P ≤ 0.001. n.s., not significant.

To investigate vaccine-induced changes in normal immune cells in the bone marrow microenvironment we re-clustered T-cell populations separately and obtained a total of 12 clusters that we identified based on differential gene expression, SingleR software analysis ^44^. and the expression of defined gene markers (Fig. 2A and Fig. S2B-C). These T-cell subpopulations were consistent across all patient samples (not shown). We analyzed changes in T-cell frequencies within each cluster, comparing paired pre- vs. post-vaccine bone marrow samples and observed statistically significant decrease in cell frequencies of naïve CD4 T cells (cluster 0) and trends toward increases in effector memory and terminal effector T cells (clusters 1 and 3, respectively) (Fig. 2B). We also performed differential gene expression analysis on each T-cell cluster, followed by pathway enrichment analysis pre- vs. post-vaccine using IPA software (Qiagen). We observed significant upregulation (adjusted log p-value > 1.3) of pathways involved in T cell activation, including TCR signaling, PI3K/AKT signaling, integrin signaling and leukocyte extravasation and down-regulation of PD-1/PD-L1 pathway following vaccination in effector T cells (Fig. 2C). Notably, there were no obvious changes in frequencies or signaling pathways in Treg (Fig. 2B cluster 9, and 2C).

To analyze the clonal composition of T cells in the microenvironment we used matched single cell TCR-seq data (Fig. 2A, right panel). A mean of 750 unique T-cell clonotypes (range 141–1596) were identified pre- and post-vaccine each for each patient. Comparing post- vs. pre-vaccine samples, we observed expansion of existing clonotypes in all patients except for two of clinical progressors LPL-005 and – 009 (Fig. 2D). Furthermore, among the 20 most prevalent clonotypes detected post-vaccination the majority increased from low frequency clonotypes that were present before vaccination, except for clinical progressors LPL-005 and – 009 (Fig. 2E). New clonotypes were also detected post-vaccination (overall 4.4%), consistent with increased T-cell clonal diversity. Increasing clonal diversity post-vaccination was also suggested in most patients, as analyzed by individual Shannon entropy scores ^45^ (Fig. 2F). Phenotypically, unique or shared T-cell clonotypes expanded in post-vaccine samples localized primarily to clusters enriched for effector memory or effector T cells (not shown). This same pattern was observed for the 20 most abundant post-vaccine clonotypes (Fig. 2G, left) with cells localized to effector memory T cells and terminal effector T cell clusters. Post-vaccine clusters also showed increased frequencies of cells in the G2-M phase of the cell cycle, consistent with increased proliferation (Fig. 2G, right). Phenotypically these top 20 post-vaccine clonotypes were primarily CD8 T cells expressing markers affecting activation, differentiation, or proliferation, including CD27, CXCR4^46^, HLA-DR^47^, PIK3RI^48^, REL^49^, and FKBP1A^50^ (Fig. 2H). In contrast, clonotypes detected only in a single T-cell or uniquely in pre-vaccine samples were distributed broadly across all T-cell subpopulations, including naïve CD4 and CD8 T cells, regulatory T cells, Th1/Th2 cells, Th17, and to lesser extent central memory T cells and effector memory phenotypes (not shown). Notably, the top 20 post-vaccine clonotypes showed a mixed pattern of up- and down-regulation of co-inhibitory molecules DUSP2 and TIGIT, respectively, with most, including PD-1, LAG3, and TIM3 (HAVCR2) showing no significant change post-vaccination (Fig. 2H). Taken together, these results suggest that vaccine therapy induced significant expansion and activation of terminal effector and effector memory T cells within the top 20 TCR clonotypes post-vaccine, with little activation of immune checkpoints.

### Tumor idiotype-specific T-cell immune responses.

To detect idiotype-specific T cell responses elicited by the vaccine treatment, we analyzed T cells isolated directly from the bone marrow tumor microenvironment. T cells were enriched from each patient’s post vaccine sample by negative selection and then stimulated with autologous immortalized normal B cells (as antigen presenting cells, APCs) transfected with either Ig VH and VL sequences (expressed as sFv’s) derived from the respective patient-specific tumor idiotype (used previously for therapeutic vaccine production), or HIV Nef as a negative control, described previously ^52^. Multiplex cytokine analysis was performed on culture supernatants. Representative post-vaccination samples are shown from patients achieving minor response, stable disease, or progressive disease clinically (Fig. 2I). All patients T cells secreted cytokines in an antigen-specific manner, with the exception of the two patients who experienced progressive disease (LPL-005 and – 009). Taken together these functional data are consistent with T-cell clonal expansion post-vaccination detected by transcriptomic analysis above.

### Vaccine-induced reduction in cross-talk between immune cell types and tumor cells in the microenvironment.

**Figure 3:**
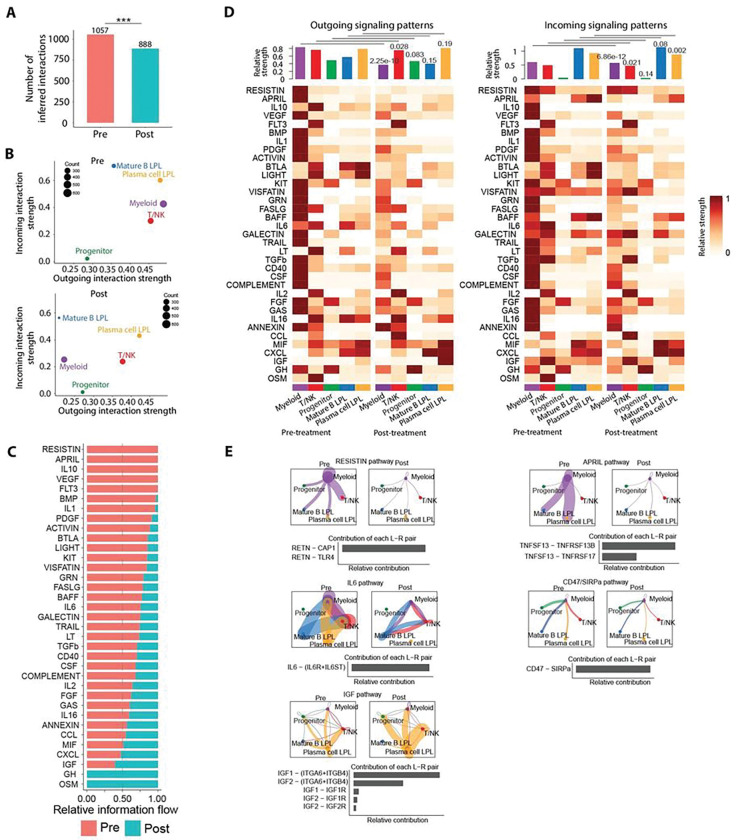
DNA vaccine significantly alters cell-cell communication networks in the tumor microenvironment. (**A**) Total numbers of ligand-receptor pair interactions in bone marrow samples pooled from all patients. Paired t-test was used to compare pre- and post-vaccine samples. ***p<0.001 (**B**) Pooled outgoing and incoming interaction strengths between the following cell types in 2D space pre- and post-treatment for all patients: LPL (mature B-lymphoid), LPL (plasma-like), myeloid, T- and NK, and normal B progenitor. Dot size indicates the number of expressed ligand-receptor pairs. Interaction strengths were calculated with Cellchat software. (**C**) Pooled relative information flows between pairwise pre- and post-vaccine datasets for all signaling pathways, sorted by increasing information flow post-treatment. (**D**) Heatmap of relative strengths of all signaling pathways pre- and post-vaccine by cell type. Outgoing and incoming signaling patterns from data pooled from all patients are shown. Paired t-test was used to compute p-values comparing signaling patterns within each cell type. (**E**) Circle plots of selected signaling pathways and relative contributions of ligand-receptor pairs. Cell types are color coded, dot size is proportional to the number of expressed ligand-receptor pairs, edge color indicates the source of outgoing signal, and edge weight is proportional to interaction strength.

To infer and analyze global changes in cell-cell communications in the tumor microenvironment after vaccination, we employed comparative CellChat ^53^ software to analyze signaling interactions among all major cell types in pre- and post-vaccination bone marrow samples. A cell-cell interaction map was constructed using aggregate sc-RNAseq data from all evaluable patients with five major interaction populations: clonal LPL mature B cells, clonal LPL plasma cell-like cells, T/NK cells, myeloid cells, and normal B progenitor cells as controls. From this cell-cell interaction map, the total number of ligand-receptor pairs contributing to communication between any two interacting cell types was analyzed. We observed that the total number of inferred interactions between the five major cell types in the tumor microenvironment significantly decreased post- compared with pre-vaccine (Fig. 3A), with this same pattern consistently observed between individual pairs of cell types (Fig. S3A-C).

To investigate which cell populations contributed to the reduction in inferred interactions, we used network centrality analysis to compare incoming and outgoing interaction strengths (Fig. 3B). Interestingly, predicted interaction strengths for myeloid and LPL mature B-cell, but not LPL plasma cell-like populations, were most dramatically reduced post-vaccine.

We then analyzed the overall information flow for multiple specific signaling pathways across the pre and post-vaccine datasets ^54^. Multiple signaling pathways were implicated as active predominantly in pre- but not post-vaccine samples, including pathways such as APRIL ^55^, which is known to promote B- or plasma cell survival, and others with known roles supporting tumor cell proliferation in solid cancers, such as RESISTIN ^56,57^, VEGF ^58^, and IL-10 ^59^, TGFβ and BMP^60^ (Fig. 3C). Moreover, the IL-6 signaling pathway, which promotes IgM secretion and LPL and plasma cell growth via the JAK/STAT pathway ^26^ was substantially reduced in post-vaccine samples.

The analysis of individual cell types revealed that myeloid cells mainly contributed to the downregulation of information flow of these signaling pathways (Fig. 3D and Fig S3D-O). For example, we observed dramatic reductions in predicted outgoing signals provided by myeloid cells for both RESISTIN and APRIL pathways, as well as IL-6, associated with their respective ligand-receptor pairs (Fig. 3E).

Paradoxically, dichotomous upregulation of the insulin-like growth factor (IGF) signaling axis post-vaccine was inferred by plasma cell, but not mature B cell LPL subpopulations, including both autocrine and paracrine pathways, consistent with a potential mechanism of escape by the former (Fig. 3, D and E). Our scRNAseq data confirmed increased expression of IGF-1 among clonal tumor cells post-vaccine in both plasma cell-like clusters (clusters 5 and 10) but not in any B-cell clusters (0, 1, and 2). We also observed an increased proportion of clonal tumor cells expressing IGF-1 in cluster 10 (Fig. S3P).

### Vaccine-induced changes in myeloid cell subpopulations in the tumor microenvironment

**Figure 4:**
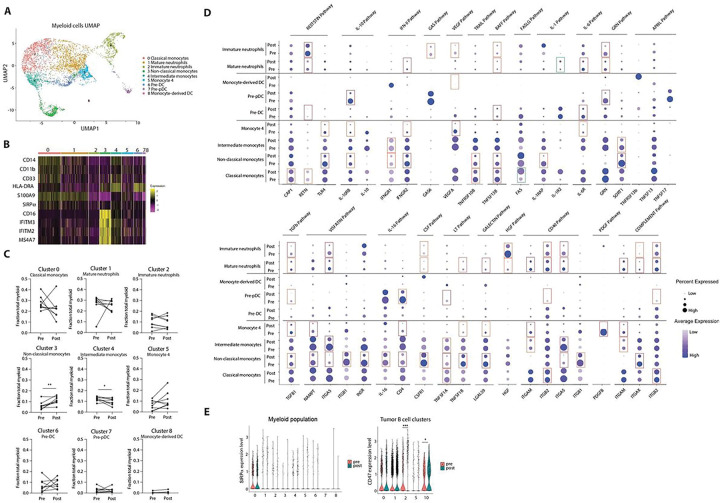
Reduced signaling by myeloid cell subpopulations post-vaccination. (**A**) UMAP of myeloid cells extracted from the total dataset. 9 clusters were generated, and cluster identities were assigned based on differentially expressed gene markers. (**B**) Heatmap of expression of selected gene markers for each cluster. (**C**) Cell frequencies pre- and post-vaccine for individual patients by myeloid cell cluster. Paired student t-test was used. (**D**) Dot plots of differentially expressed genes pre- and post- vaccine in selected pathways identified in Fig. 3 by cluster. Pooled data from all patients. Significantly downregulated or upregulated genes in post-vaccine samples are highlighted by red and green rectangles respectively. Color intensity and dot size correspond to expression level and relative proportion of positive cells, respectively. (**E**) Violin plots of SIRPa and CD47 gene expression pre- and post-vaccination by cell cluster in myeloid and B-cell tumor populations, respectively. Pooled data from all patients. Two-sided Wilcoxon test was used. *P ≤ 0.05, ***P ≤ 0.001. n.s., not significant.

Given that vaccination was associated with significantly reduced cell-cell communication patterns in the tumor microenvironment, most pronounced in outgoing signals provided by myeloid cells to clonal LPL cells, we further analyzed subpopulations of myeloid cells by re-clustering them based on differential gene expression analysis from the combined datasets of pre- and post-vaccine bone marrow cells from all patients. We obtained a total of 9 clusters, based on the differential expression of established marker genes (Fig. 4. A, B and fig. S4A). Myeloid cell populations were consistent across all individual patient samples (not shown).

We analyzed changes in cell frequencies per cluster in paired pre- vs. post-vaccine patient samples and observed significant increases in frequencies of CD14^−^CD16^+^ non-classical monocytes (cluster 3) and concomitant significant decreases in the frequencies of CD14^+^CD16^+^ intermediate monocytes (cluster 4, Fig. 4C). Given that monocyte differentiation is believed to proceed from classical CD14^+^CD16^−^ to non-classical CD14^−^CD16^+^ monocytes via intermediate CD14^+^CD16^+^ monocytes ^61^, these results may suggest that monocytes in the tumor microenvironment post vaccination undergo increased differentiation from intermediate to non-classical subpopulations, thereby causing a general skewing away from classical monocytes. This hypothesis was also supported by the trend towards decreasing CD14^+^CD16^−^ classical monocyte frequencies (cluster 0) observed post- vs. pre-vaccination, although these differences did not reach statistical significance.

Next, we sought to identify the specific myeloid cell clusters which contributed to the dramatic reductions in outgoing predicted signals provided by myeloid cells to LPL cells by analyzing each of the individual signaling pathways in Fig. 3D. Comparing post- vs. pre-vaccine samples, most of the signaling pathways affected were associated with reductions in monocytic subpopulations, particularly cluster 3 non-classical monocytes, and to a lesser extent cluster 0 classical monocytes (Fig. 4D). Changes were also observed across many of the pathways for cluster 1 of mature neutrophils, but these were generally of lesser magnitudes.

Taken together, these data suggest that vaccination was associated with clear reductions in pro-tumoral outgoing signals provided by non-classical monocytes to LPL cells, but with a paradoxical expansion of this myeloid subpopulation.

Finally, because of the availability of potential therapeutic intervention, we also performed cell-cell communication analysis of the CD47-SIRPα pathway which predicted overall decreased signaling after vaccination (Fig. 3E), despite increased CD47 expression on at least one lymphoid (B-cell cluster 2) and one plasmacytoid (B-cell cluster 10) LPL tumor subpopulation. SIRPα was observed on cluster 0 classical monocytes pre-vaccination, with no significant change in expression post-vaccination (Fig. 4E).

## Discussion

In the absence of any standard treatment for patients with smoldering phase LPL/WM, patients are typically managed by active surveillance alone. The median time to progression to the symptomatic phase is 3.9 years ^62^. Early intervention with a well-tolerated therapeutic agent, such as a vaccine, that could delay progression to symptomatic phase disease, would therefore be highly desirable. The individualized therapeutic DNA vaccines used in this trial appear to be safe, with no patients experiencing dose limiting toxicities (DLTs) or Grade 4 adverse events (AEs). Most of the patients experienced potential clinical benefit, with documentation of stable disease or better for a median of 61 + months, including one patient who achieved a minor response (MR).

The reasons for lack of more robust objective clinical responses were revealed by direct interrogation of the tumor microenvironment by single-cell transcriptome analysis. Comparing paired pre- and post-vaccine bone marrow samples available from eight of the nine patients, we observed a striking dichotomous pattern of significantly reduced numbers of clonal tumor idiotype-expressing B-cells post-vaccine in the majority of patients, but no change in clonally related plasma cell-like clusters of any patient (Fig. 1D). Heterogeneity within LPL, consisting of separate mature B-cell and plasma cell-like subpopulations, has been described ^63,64^. In our dataset, plasma cell-like cells were detected at lower frequencies, compared with mature B cell-like subpopulations, but partial loss of this fragile cell subpopulation during frozen sample preparation cannot be ruled out ^40^. Furthermore, there was an associated pattern of downregulation of expression of genes known to be essential for lymphoma cell proliferation and protein synthesis in tumor clusters of mature B cells, but not of plasma cell-like LPL subpopulations (Fig. 1F). Pathway analysis predicted global downregulation of signaling pathways known to be critical for B-cell survival and conversely, upregulation of pathways known to promote tumor cell apoptosis and other forms of cell death, in mature B-cell, but not plasma cell-like LPL clusters, consistent with a vaccine-induced antitumor response against the mature B-cell LPL compartment.

Downregulation of expression of HLA molecules, particularly class II, has been recognized as one immune-evasion mechanism in cancer^39,40^. Indeed, we observed that plasma and plasmablast-like LPL cells expressed low levels of HLA class II genes. This represents a potentially intrinsic mechanism of resistance by plasma cell-like subpopulations, as low levels were observed in both pre- and post-vaccine samples (Fig. 1G). In contrast, the trend towards downregulation HLA class II gene expression observed in LPL cluster 2 cells post-vaccine, compared with pre-vaccine, suggests a potential mechanism of immune evasion among mature B-cell subpopulations (Fig. 1G and H). In contrast, dichotomous upregulation of the IGF signaling axis post-vaccine was inferred by plasma cell, but not mature B cell LPL subpopulations (Fig. 3D and E), suggesting a possible mechanism of acquired resistance to vaccine therapy. The IGF axis has been implicated in acquired drug resistance in various hematologic cancers, and selective IGF-1 receptor inhibitors could block tumor cell proliferation and migration and overcome resistance to treatment of multiple myeloma, and lymphomas with bortezomib, EZH2 inhibitors and crizotinib ^66–68^. Furthermore, our finding that the proportion of tumor cells expressing IGF-1 was also increased in one of two plasma cell-like clusters post-vaccine suggests that clonal selection of IGF signaling-dependent tumor clones cannot be excluded (Fig. S3P).

The chemokine-antigen fusion vaccine platform was designed to elicit robust T-cell immunity, by targeting idiotype antigen delivery to chemokine receptors on antigen presenting cells ^10,11^. Indeed we observed that vaccine therapy induced dynamic changes in T cells in the tumor microenvironment, consistent with generation of antigen-specific immune responses. Trends toward increases in effector memory and terminal effector phenotypes post-vaccine (Fig. 2B and H) were associated with upregulation of pathways involved in T-cell activation (Fig. 2C), expansion of individual T-cell clonotypes (Fig. 2D, E), increased T-cell clonal diversity (Fig. 2F), and functional LPL idiotype-specific cytokine production (Fig. 2I).

Tracking the top 20 TCR clonotypes pre- and post-vaccine suggested that they expanded from pre-existing idiotype-specific effector/effector memory cells (Fig. 2G), rather than naïve cells primed by vaccine. Recent studies have suggested that Treg cells create an immunosuppressive milieu in WM, the most common subtype of LPL ^69^. However, we observed no obvious changes in frequencies or signaling pathways in Treg that would suggest an effect of vaccination on this subpopulation (Fig. 2B cluster 9, and 2C). T cells in the microenvironment also showed a mixed pattern of up- and down-regulation of co-inhibitory pathways (Fig. 2C), with most showing no significant change post-vaccination (Fig. 2H). Taken together, these results suggest little activation of co-inhibitory molecules by vaccine therapy.

Our vaccine also globally altered the levels of the cell-cell communication networks and signaling strength across various other cell populations in the tumor microenvironment. We detected significant downregulation of signaling pathways post-vaccine that likely directly promote growth of LPL cells, such as APRIL and IL-6 which are known to promote B- or plasma cell survival (Fig. 3C to E). Other pathways were reduced post-vaccine, such as RESISTIN, which has a known role in supporting proliferation of solid cancers by binding CAP1 receptors. A role for RESISTIN in supporting LPL has not been previously inferred, but it induced multidrug resistance in human multiple myeloma ^56^.

Bioinformatic analysis also identified a predominant role for myeloid cells in the tumor microenvironment as a source of vaccine-induced, downregulated pro-tumoral signaling to LPL cells. The global signaling pathways affected were primarily associated with monocytic, rather than granulocytic or dendritic cell subpopulations, particularly non-classical CD14^−^CD16^+^ monocytes, and to a lesser extent classical CD14^+^CD16^−^ monocytes (Fig. 4D). Myeloid cells have been recognized as key component of the immune suppressive microenvironment in solid tumors and this observation has been extended more recently to B-cell tumor microenvironments ^70^. The trend we observed towards reduction of classical monocytes may be of particular relevance, as this subpopulation has been recently shown to pro-tumoral in multiple myeloma ^71^. Additionally, our gene signature analysis revealed that this cluster may contain myeloid derived suppressor cells (MDSC) which have also been extensively characterized as immune-suppressive (Fig. S4B, *57*)). Finally, reports indicate that an increased pro-inflammatory myeloid signature is an early step in the development of WM and in monoclonal gammopathy of undetermined significance (MGUS) ^72^.

One potential strategy to further overcome resistance to myeloid signaling may be therapeutic blockade of SIRPα-CD47, an emerging checkpoint utilized by cancer cells to evade immune responses. Despite increased expression of CD47 (“do not eat me” signal) on at least one mature B-cell- (cluster 2) and one plasma cell-like (cluster 10) LPL tumor subpopulation after vaccination, CellChat analysis predicted overall decreased signaling to SIRPα on myeloid cells which expression was confirmed on classical monocytes (Fig. 4E). Taken together, these results suggest that vaccine therapy was associated with significant reduction of pro-tumoral signaling by myeloid cells in the LPL microenvironment.

Recent improvements in bioinformatics, design, and manufacturing are facilitating the clinical development of individualized neoantigen cancer vaccines. As a prototype, our idiotype neoantigen vaccine demonstrated safety, the ability to significantly reduce clonal mature B-cell, but not plasma cell-like, LPL subpopulations and to favorably perturb the tumor microenvironment. Future functional studies of the pathways affected are needed to confirm mechanisms of resistance elucidated and to design combination strategies to circumvent them. Such strategies could include adding IFNγ or epigenetic drugs, designed to increase HLA molecule expression on plasma cell-like LPL subpopulations ^73^ and combining neoantigen vaccines with agents that specifically target plasma cells, such as anti CD38 antibodies ^74^, or pathways known to promote their growth, such as IGF-1 receptor inhibitors ^75^. Furthermore, our data suggest that combinations of these vaccines with myeloid cell checkpoint blockade may be worthwhile. Finally, although little activation of co-inhibitory molecules was observed by vaccine therapy, elevated PD-1 ligands on human WM cells and exhausted CD8 T cells in the WM microenvironment have been reported by others ^76^, suggesting that there may still be a role for therapeutic T-cell checkpoint blockade combined with this vaccine.

## Materials and Methods

### Experimental Design

This was an open-label phase I trial conducted at the University of Texas M.D. Anderson Cancer Center. Patients were enrolled between March 2015 and August 2017. Patients received a series of three DNA vaccinations with autologous MIP-3α fused lymphoma idiotype at 4-week intervals intradermally into both thighs by needle-free injection device (PharmaJet, Golden, CO). Consecutive patients were enrolled to dose cohorts 1 (500 μg) and 2 (2500 μg) according to a standard 3 + 3 statistical design.

The primary objective was to evaluate the safety profile of the vaccine and to determine its maximum tolerated dose (MTD). Clinical laboratory testing, patient reporting, and physical examination findings were used to evaluate adverse events (AEs), including serious adverse events (SAEs). Toxicities were graded according to the NCI Common Toxicity Criteria v4.0. Dose limiting toxicity (DLT) was defined as a ≥ grade 2 allergic reaction, ≥ grade 2 autoimmune reaction, and any grade 3 or 4 toxicity except for fever, grade 4 fever which subsequently required 50% dose reduction. MTD was defined as the highest dose level in which 6 patients have been treated with less than 2 instances of DLT.

Initial disease response was assessed one month after the final vaccination according to International WM consensus panel response criteria from the 6th International Workshop^77^.

### Patients

Eligible patients had a diagnosis of smoldering lymphoplasmacytic lymphoma (LPL) confirmed by tissue diagnosis, with a monoclonal heavy and light chain as determined by flow cytometry. All participants were required to be able to provide informed consent. Patients were excluded if they had a history of autoimmune diseases except for Hashimoto’s thyroiditis, or either a positive antinuclear antibody titer or anti-double strand DNA titer. Conduct of this trial was approved by the University of Texas M.D. Anderson Cancer Center institutional review board (protocol 2009 – 0465) and was carried out in accordance with the Declaration of Helsinki and the International Conference on Harmonization Guidelines for Good Clinical Practice.

### Bone marrow aspirates and cryopreservation

All patients had up to 10 ml of bone marrow aspirated before treatment for morphological sorting, immunophenotyping, and characterization. A second 15 ml bone marrow aspirate sample was obtained from the contralateral side for additional tumor cell banking for vaccine production and/or correlative analysis. Bone marrow mononuclear cells (BMMNCs) were isolated by density gradient centrifugation using Ficoll. Mononuclear cells were washed three times with 45 mL PBS (800g), counted, and viably cryopreserved in 10% DMSO.

### Generation of individualized DNA vaccines

LPL B cells generally comprise > 30% of the total B-lymphocyte population in bone marrow ^78^. The unique lymphoma idiotype for each patient’s tumor was identified based on the clonal amplification of a predominant Ig heavy and light chain V(D)J sequence ^12^. MIP-3α fused lymphoma idiotype plasmid DNA vaccines were prepared from each patient’s bone marrow as described previously ^79^. The plasmid DNA was then amplified and purified from *E. coli* according to Good Manufacturing Practices (GMP) standards by FUJIFILM Diosynth Biotechnologies U.S.A., Inc., (College Station, TX).

#### Bone marrow sample processing for single cell RNA sequencing.

Cryopreserved bone marrow samples pre and post vaccine were processed together for each patient. Cells were thawed at 37°C and resuspended in culture media. Dead cell removal was performed using StemCell EasySep Annexin V kit (Cat#17899). Cell were resuspended in PBS with 0.04% BSA and count and viability was determined using automated cell counted (BioRad) prior to loading onto 10x Genomics Chip (Chromium Single Cell 5’ Kit). LPL-008 pre and post samples after thawing, as described above, were used for staining with hashtag antibodies (Biolegend TotalSeq-C0251, Cat #394661 and TotalSeq-C0252, Cat #393663) according to manufacturer’s protocol. Pre and post vaccine samples were mixed in a 1:1 ratio and loaded onto the 10x Genomics chip. Libraries were prepared using Chromium Single Cell 5’ Kit (10x Genomics) for gene expression, TCR and BCR and QC was performed using Agilent High Sensitivity DNA kit on the Agilent 2100 bioanalyzer. Libraries were sequenced on Illumina HiSeq 2500 (Read1: 26 cycles, i7 index: 8 cycles, Read2: 101 cycles, Sequencing depth: 20,000/ read pairs per cycle for gene expression, Read1: 151 cycles, i7 Index: 8 cycles, Read2: 151 cycles, sequencing depth: 5,000/ read pairs per cell for TCR/BCR). Raw sequencing reads were processed using CellRanger pipeline using default settings (10x Genomics, software version 3.1.0).

#### Single cell RNA-seq data analysis.

Filtered gene expression matrices generated by CellRanger pipeline were used for downstream analysis using Seurat package (version 3) ^81,82^. Loupe Browser was used to resolve Hashtag identities of cellular barcodes (10x Genomics version 3). Cells were filtered based on total mRNA counts, total genes detected, and a mitochondrial content. We obtained an average of 2163 cells per sample (range 830–4680), totaling 36,777 cells in the dataset. Dataset were normalized and 2000 most variable genes were selected. Datasets were merged sequentially using IntegrateData function in Seurat. To improve clustering resolution, a dataset “A single cell immune cell atlas of human hematopoietic system” from Human Cell Atlas website (https://data.humancellatlas.org/) was obtained. Dataset file was subset to obtain samples of normal healthy bone marrow of 15 oldest individuals available based on metadata files, subject to identical filtering and processing, followed by sequential data integration as above. Feature scaling, PCA, clustering and UMAP analysis were performed on merged datasets using integrated assay. Identification of markers of cell populations were done with FindAllMarkers function. A total of 25 clusters were identified (Fig. S1B-F) which were consistent across all patient samples (Fig. S1D). For single cell BCR and single cell TCR sequencing, filtered contig annotation CellRanger output files were processed with custom R script and were added to the Seurat object as metadata based on cell barcodes (Fig. S1E). For further sub-analysis, B cell, T cell and myeloid cell populations were separated out using subset function and re-analyzed with PCA, clustering and UMAP as described above. Differential gene expression pre vs post vaccine was performed using FindMarkers function with default parameters. Volcano plots were created using EnhancedVolcano package^84^ Pathway analysis and biological processes analysis was performed using IPA software (Qiagen) (adjusted log p-value > 1.3) and visualized using R. Heatmaps, dot plots and violin plots were generated with Seurat. TCR clonotype analysis was performed using immunarch R package according to instructions^85^.

#### Bone marrow T cell stimulation and cytokine 30-Plex human panel.

Cells were thawed at 37°C and washed with ImmunoCult-XF T cell media (StemCell Cat#10981) supplemented with 1U/mL of DNAse (Thermo Sci Cat#EN0521). Cells were centrifuged and resuspended in EasySep buffer (StemCell Cat#20144), followed by exposure to CD20 magnetic microbeads (Miltenyi Biotec Cat#130-0910104). T cells were enriched by negative selection (EasySep Cat#10981) and resuspended in T cell media supplemented with IL-2 at 50U/mL (clinical grade), IL-7 at 25ng/mL (Miltenyi Biotec, Cat#130-095-361) and IL-15 at 25ng/mL (Miltenyi Biotec, Cat#130-095-762) to a concentration of 2 ×10^6^ cells/mL. 125ul of cell suspension were plated in V-bottom 96-well plates. Autologous patient derived immortalized B cells were generated as described previously ^52,83^. Cells were suspended in PBS, irradiated with 1500 rads for 5min, spun down and resuspended in Neon Buffer R (Invitrogen). mRNA encoding the patient’s unique patient Idiotype, scFv-MITD or irrelevant antigen (Nef-MITD) were prepared as described previously^52^and electroporation was performed using Neon MPK5000 system (Invitrogen) with settings: pulse voltage: 1150 V, pulse width: 30 ms, pulse number: 2. Electroporated APC were resuspended in T cell media supplemented with IL-2, IL-7 and IL-15 to a final concentration of 2×10^6^ cells/mL and 125ul of cells were added to V-bottom 96-well plate containing T-cell suspensions. Cells were co-cultured in 37°C incubator for a total of 10 days. At day 3 and 7, 125ul of culture media was replaced with fresh T cell media supplemented with IL-2, IL-7 and IL-15 at 50U/mL, 25ng/mL and 25ng/mL for day 3 and 100U/mL, 25ng/mL and 25ng/mL on day 7 respectively. On day 10 cells were washed in 500 μL of PBS, spun down and supernatants were snap-frozen at −80°C. Supernatants were used for 30-Plex human panel analysis according to manufacturer’s protocol.

### Cell–cell communication analysis

The CellChat package was used to infer cell–cell communications between the following cell types via interaction-network analysis: LPL (mature B-lymphoid), LPL (plasma cell-like), myeloid, T- and NK, and normal B progenitor cells. A Seurat object was used as input for CellChat following standard protocols (https://github.com/sqjin/CellChat.NetAnalysis_signalingRole_heatmap) and was used to compute the comparison of overall signaling pathways in pre- vs. post-vaccine samples. Circle plots were generated via netVisual_aggregate, vertex.size = groupSize, respectively. In circle plots, edge color indicates the source of outgoing signal, and edge weight is proportional to interaction strength. Incoming and outgoing strength were calculated via CellChat function with default parameter. The information flow of each signaling pathway was defined by the sum of communication probabilities among all pairs of cell groups in the inferred network and was generated via CellChat rankNet. Statistical comparisons of relative interaction strengths were performed by Student’s t-test

## Figures and Tables

**Table 1:** Baseline Patient Characteristics

Characteristic (range)	Cohort 1 500μg (n = 3)	Cohort 2 2500μg (n = 6)
Median Age	65 (56–71)	69 (61–78)
Male Sex	100%	67%
ECOG performance status 0–1	100%	100%
Time from diagnosis of SWM to 1 st vaccination (yrs.)	8.1 (1.4–8.8)	2.0 (0.7–10.8)
Genotype* (no. of patients)		
MYD88WT/CXCR4WT	1	1
MYD88 L265P/CXCR4WT	2	3
MYD88 L265P/CXCR4WHIM	0	1
Bone Marrow infiltration (%)	30 (25–40)	30 (10–50)
Serum IgM (mg/dL)	2900 (814–3150)	3255 (473–7210)
Monoclonal Protein (g/dL)	2.3 (0.9–2.8)	2.5 (0.4–6.3)
Hemoglobin (g/dL)	13.6 (12.2–14.9)	12.3 (137–353)
Platelet count (K/μL)	204 (189–372)	278 (137–353)
Beta 2 microglobulin (mg/L)	2.5 (2.0–3.7)	2.5 (1.7–3.7)
Albumin (g/dL)	77.7 (76.9–80)	4.1 (3.7–4.4)
LDH (U/L) (normal range: 313–618)**	378 (376–405)	337 (201–613)

* Genotype not available for 1 patient in Cohort 2

* LDH not available for 1 patient in Cohort 2

**Table 2:** Adverse Events

	Total No. of patients (n = 9)	
Most common adverse events	Any Grade	≥ Grade 3
Hematologic		
Leukocytopenia	6	
Anemia	4	
Gastrointestinal		
Nausea	5	
Diarrhea	3	
General		
Fatigue	4	
Myalgia	3	
Respiratory		
Dyspnea	3	
Pleural Effusion	1	1
Cardiac		
Pericardial Effusion	1	
Dermatologic		
Injection Site Reaction	3	
Lab Abnormalities		
Creatinine increase	4	
Hyperglycemia	6	
